# Expression of Endogenous Epitope-Tagged GPR4 in the Mouse Brain

**DOI:** 10.1523/ENEURO.0002-24.2024

**Published:** 2024-03-06

**Authors:** Elizabeth C. Gonye, Alexandra V. Dagli, Natasha N. Kumar, Rachel T. Clements, Wenhao Xu, Douglas A. Bayliss

**Affiliations:** ^1^Department of Pharmacology, University of Virginia, Charlottesville, Virginia 22903; ^2^School of Biomedical Sciences, University of New South Wales Sydney, New South Wales 2052, Sydney, Australia; ^3^Genetically Engineered Mouse Model Core, University of Virginia, Charlottesville, Virginia 22903

**Keywords:** CRISPR/Cas9, epitope tag, GPR4, knock-in

## Abstract

GPR4 is a proton-sensing G-protein-coupled receptor implicated in many peripheral and central physiological processes. GPR4 expression has previously been assessed only via detection of the cognate transcript or indirectly, by use of fluorescent reporters. In this work, CRISPR/Cas9 knock-in technology was used to encode a hemagglutinin (HA) epitope tag within the endogenous locus of *Gpr4* and visualize GPR4-HA in the mouse central nervous system using a specific, well-characterized HA antibody; GPR4 expression was further verified by complementary *Gpr4* mRNA detection. HA immunoreactivity was found in a limited set of brain regions, including in the retrotrapezoid nucleus (RTN), serotonergic raphe nuclei, medial habenula, lateral septum, and several thalamic nuclei. GPR4 expression was not restricted to cells of a specific neurochemical identity as it was observed in excitatory, inhibitory, and aminergic neuronal cell groups. HA immunoreactivity was not detected in brain vascular endothelium, despite clear expression of *Gpr4* mRNA in endothelial cells. In the RTN, GPR4 expression was detected at the soma and in proximal dendrites along blood vessels and the ventral surface of the brainstem; HA immunoreactivity was not detected in RTN projections to two known target regions. This localization of GPR4 protein in mouse brain neurons corroborates putative sites of expression where its function has been previously implicated (e.g., CO_2_-regulated breathing by RTN) and provides a guide for where GPR4 could contribute to other CO_2_/H^+^ modulated brain functions. Finally, GPR4-HA animals provide a useful reagent for further study of GPR4 in other physiological processes outside of the brain.

## Significance Statement

GPR4 is a proton-sensing G-protein-coupled receptor whose expression is necessary for a number of diverse physiological processes including acid-base sensing in the kidney, immune function, and cancer progression. In the brain, GPR4 has been implicated in the hypercapnic ventilatory response mediated by brainstem neurons. While knock-out studies in animals have clearly demonstrated its necessity for normal physiology, descriptions of GPR4 expression have been limited due to a lack of specific antibodies for use in mouse models. In this paper, we implemented a CRISPR/Cas9 knock-in approach to incorporate the coding sequence for a small epitope tag into the locus of GPR4. Using these mice, we were able to describe GPR4 protein expression directly for the first time.

## Introduction

GPR4 is a class A G-protein-coupled receptor (GPCR) and a member of the proton-sensing subfamily of GPCRs that also includes TDAG8 (*Gpr65*), OGR1 (*Gpr68*), and G2A (*Gpr132*; Ludwig et al., 2003; Rowe et al., 2021; Sisignano et al., 2021). GPR4 has been implicated in both peripheral and central physiological functions. It plays a role in angiogenesis (Kim et al., 2005; Qiao et al., 2006; Yang et al., 2007; Ren et al., 2016; Miltz et al., 2017; Ouyang et al., 2021; Li et al., 2023), monocyte migration (Huang et al., 2007; Chen et al., 2011; Dong et al., 2013), chronic inflammation (Miltz et al., 2017; Sanderlin et al., 2017, 2019; Krewson et al., 2020; Liu et al., 2020; Marie et al., 2023), ischemia/reperfusion injury (Dong et al., 2017), maintenance of acid–base balance by the kidney (Yang et al., 2007; Sun et al., 2010, 2015; Cheval et al., 2021), and cancer cell migration/metastases (Sin et al., 2004; Castellone et al., 2011; Wyder et al., 2011; Jing et al., 2016; Yu et al., 2019; Xue et al., 2020; Marie et al., 2023). In the central nervous system, GPR4 has been associated with central respiratory chemosensitivity, contributing both to an atypical CO_2_/H^+^-dependent vasoconstriction in brainstem regions associated with CO_2_-regulated breathing and to direct modulation by CO_2_/H^+^ of putative respiratory chemosensory neurons in the retrotrapezoid nucleus (Kumar et al., 2015; Hosford et al., 2018; Wenzel et al., 2020).

Detection of GPR4 within the mouse, including in the brain, has proven technically challenging due to a lack of specific antibodies. Previous experiments examining GPR4 localization in the mouse have indirectly inferred sites of expression using a GPR4 promotor-driven fluorescent marker (Sun et al., 2016; Sanderlin et al., 2017; Marie et al., 2023), Cre expression from the GPR4 locus to enable fluorescent marker lineage tracing (Hosford et al., 2018) or relied on RNA detection as a proxy for protein expression (An et al., 1995; Mahadevan et al., 1995; Wyder et al., 2011; Kumar et al., 2015; Shi et al., 2017; Hosford et al., 2018). These methods each have limitations: they either do not reflect GPR4 expression at the time of tissue harvest or do not inform subcellular localization of GPR4 itself.

To circumvent these technical limitations, we pursued an endogenous knock-in strategy to incorporate a small hemagglutinin (HA) tag into GPR4 using CRISPR/Cas9 genome editing. With this approach, we used well-characterized, highly specific, and easily accessible epitope antibodies to characterize GPR4 expression in the mouse brain. We compare protein and RNA distribution and provide new quasi-quantitative information on regional and subcellular localization of GPR4 protein in different cell populations.

## Materials and Methods

### Animal care

This study was completed in accordance with the requirements of the Institutional Animal Care and Use Committee at the University of Virginia and was completed in an AALAC-accredited animal care facility. All efforts were made to minimize the number of animals used in these studies. Tissue used in this study was obtained from 8 to 12-week-old C57/BL6 mice (for in situ studies, *n* = 3, 2 male, 1 female) or mixed B6SJL background animals (for immunohistochemical studies: *n* = 6, 3 male, 3 female). Animals were group housed (3–5 animals per cage) on cob bedding with *ad libitum* access to food, water, and enrichment items. Cage bedding, food, and water were changed weekly. The animal housing facility was maintained on a 12 h dark/light cycle at 22°C and humidity 50%. Animals were monitored daily by veterinary staff for distress or injury.

### GloSensor cAMP assay

HEK293T cells were plated in a poly-ʟ-lysine coated white 96-well plate (Greiner Bio-One 655074) at a density of 5 × 10^4^ cells per well in high glucose DMEM (Invitrogen 11965-092) with 1× sodium pyruvate (Invitrogen 11360-070) and 10% heat-inactivated fetal bovine serum. Cells were allowed to incubate overnight at 37°C/5% CO_2_. The following day, cells were transfected with the GloSensor-22F cAMP plasmid (Promega E2301) and wild-type GPR4 (Ludwig et al., 2003), DRY-mutant G-protein-binding deficient GPR4(R117A) (Kumar et al., 2015), or C-terminal HA-tagged mouse GPR4 (generated from wild-type GPR4 construct using overlap extension primer sequences: CTGCTGCCCCCGGCACAGGGATCCTCAGGTTACCCATACGATGTTCCAG and GGCGCGGTCTAGACTATC AAGCGTAATCTGGAACATCGTATG) in pcDNA3.1 (final concentration, 0.02 ng/µl). Constructs were mixed with Lipofectamine 2000 (ThermoFisher 11668027) and added to cells according to manufacturer instructions and allowed to incubate for 20 h. The next day, transfection media was removed and replaced with HBSS (Invitrogen 14175-095) containing 2% v/v GloSensor Reagent (Promega E1290). Cells were equilibrated for 2 h at 37°C/5% CO_2_. After equilibration, GloSensor reagent solution was replaced with HBSS at different pH or containing 10 µM forskolin (Sigma F3917) as a positive control and incubated for 20 min at room temperature. Luminescence was detected using a Biotek Synergy HTX plate reader. Data were normalized to pH 8.0 HBSS as baseline and 10 µM forskolin in pH 8.0 HBSS as ceiling.

### Generation of *Gpr4*^HA^ knock-in mouse model

CRISPR-assisted genome editing technology was used by the Genetically Engineered Murine Model (GEMM) Core at the University of Virginia to generate the *Gpr4*^HA^ knock-in mouse. In short, an sgRNA (CATGGGGCTCACTGTGCCGGGGG) was selected based on a search through the coding region of the C terminus of *Gpr4* using the CRISPR guide design algorithm CRISPOR (http://crispor.tefor.net/). The HA tag (YPYDVPDYA-STOP, TATCCATACGACGTTCCAGATTACGCTTAG) preceded by a Gly-Ser-Ser-Gly (GGATCCTCAGGT) linker was introduced onto the C-terminal sequence of the wild-type (WT) *Gpr4* gene to generate the *Gpr4*-HA donor (199mer ssODN, sequence below). The sgRNA, Cas9, and ssODN were purchased from Integrated DNA Technologies. The sgRNA was diluted to 200 μM in RNase-free microinjection buffer (10 mM of Tris-HCl, pH 7.4, 0.25 mM of EDTA). Ribonucleic protein (RNP) complex was formed by mixing and incubating Cas9 at 0.2 ug/µl with sgRNA at 3 μM in RNase-free microinjection buffer at 37°C for 10 min. The ssODN incorporating the epitope tag was added at a concentration of 0.3 ug/µl. Fertilized eggs were collected from B6SJLF1 females mated with males of the same line (The Jackson Laboratory). The RNP/ssODN were codelivered by electroporation with a NEPA21 super electroporator (Nepa Gene) into the fertilized eggs, which were cultured overnight in KSOM medium (EMD Millipore) at 37°C in 5% CO_2_. The next morning, zygotes that had reached the two-cell stage were implanted into the oviducts of pseudopregnant foster mothers of the ICR strain (Envigo). Pups born to foster mothers were screened using tail snip DNA for PCR genotyping followed by Sanger sequencing, with analysis of the knock-in performed using the Synthego Inference of CRISPR Edits (ICE) Analysis tool (https://ice.synthego.com). Germline transmission of the desired allele was confirmed by breeding the founders with wild-type C57BL/6J mice (The Jackson Laboratory) and sequencing the pup DNA, as described above. Homozygous *Gpr4*^HA/HA^ and littermate *Gpr4*^+/+^ controls were used for plethysmography and immunohistochemistry experiments; although immunostaining was observed with *Gpr4*^HA/+^ heterozygotes in regions of high expression (e.g., RTN), the labeling was less intense in neuronal somata and more difficult to discern in neuronal processes.

ssODN sequence:

CGTCCGGGGCTGTCTGGGCAGTGCCTCCGACTGCCCAGGGGGACCAGGTGCCACTGAAGGTGCTGCTGCCCCC GGCACAGGGATCCTCAGGTTATCCATACGACGTTCCAGATTACGCTTAGGCCCCATGCCCAACTGTCATCCTGCACC CTTCCGGTTGTATGCAAATGTGTGTAAATATGTCCATGTGAATTACAAG.

### Whole body plethysmography

Ventilatory responses were measured in conscious mice by whole body plethysmography in chambers manufactured by Data Sciences International (601-1540-001) and recorded with IOX software (EMKA Technologies). A mass flow regulator provided quiet, constant and smooth flow through the animal chamber (0.5 L/min). Mice were familiarized with the plethysmography chamber the day prior to testing (3–4 h acclimation period) and again immediately before the testing protocol (for at least 2 h). The typical protocol entailed three sequential incrementing CO_2_ challenges (7 min exposures to 4%, 6%, 8% CO_2_, balance O_2_; each separated by 5 min of 100% O_2_). Hypercapnic exposure was performed in hyperoxia to minimize contributions of peripheral chemoreceptors to the hypercapnic ventilatory reflex and attribute ventilatory effects to central chemoreception. CO_2_ tension in the chambers was verified with a capnograph. After data collection, Poincare analysis of the breathing frequency over the final 3 min of each challenge period for CO_2_ was performed to select periods of regular, calm breathing for analysis of frequency, tidal volume, and minute ventilation.

### Immunohistochemistry

Mice were anesthetized with ketamine/xylazine (200/14 mg/kg, i.p.), perfusion-fixed (4% PFA/0.1 M PB) and sectioned using a vibratome (30 μm sections, 1:3 serial). Sections were stored at −20°C in cryoprotectant solution consisting of the following: 0.05 M sodium phosphate buffer (PB), 30% ethylene glycol, 20% glycerol. All primary and secondary antibodies used in this study are listed in Table 1. Upon removal from cryoprotectant solution, sections were washed in 0.1 M PB (3 × 5 min) and then Tris saline (TS), pH 7.4 (3 × 5 min). Sections were blocked in TS containing 0.3% Triton X-100 and 10% fetal horse serum (FHS) at room temperature for 45 min. Sections were then incubated in primary antibody solution (TS/0.1% Triton X-100/1% FHS) overnight at 4°C with gentle rocking. After primary antibody incubation, sections were washed in TS (3 × 10 min) before incubation for 90 min at room temperature in secondary antibody solution (TS). DAPI solution was added during the last minute of secondary antibody incubation period. Sections were mounted on Superfrost Plus glass slides (Fisher Scientific 12-550-15) and sealed with ProLong Gold antifade reagent with DAPI (Invitrogen P36935) before imaging on a Zeiss AxioImager Z1 widefield epifluorescence microscope (Figs. 3, 5, 7, 8) or a Zeiss LSM700 scanning confocal microscope (Figs. 2, 9, 10).

**Table 1.** Antibodies

Antibody	Product number	RRID	Dilution
Goat anti-ChAT	Millipore AB144P	AB_2079751	1:200
Mouse anti-TPH	Sigma T0678	AB_261587	1:250
Rat anti-PECAM	Synaptic Systems HS-351 117	AB_2619721	1:500
Goat anti-PHOX2B	R&D Systems AF4940	AB_2861427	1:100
Rabbit anti-HA (C29F4)	Cell Signaling Technologies #3724	AB_1549585	1:2,000
Rat anti-mCherry	ThermoFisher M11217	AB_2536611	1:2,000
Goat anti-CGRP	Abcam ab36001	AB_725807	1:1,000
Donkey anti-mouse Alexa488	Jackson Immuno 715-546-150	AB_2340849	1:500
Donkey anti-rabbit Cy3	Jackson Immuno 711-166-152	AB_2313568	1:500
Donkey anti-goat Cy3	Jackson Immuno 705-166-147	AB_2340413	1:500
Donkey anti-goat Alexa647	Jackson Immuno 705-606-147	AB_2340438	1:500
Donkey anti-rat Cy3	Jackson Immuno 712-166-150	AB_2340668	1:500
Donkey anti-rat Alexa647	Jackson Immuno 712-606-150	AB_2340695	1:500

### RNAscope in situ hybridization

Fresh fixed tissue sections brainstem/midbrain (30 µm) or forebrain (40 µm) were mounted on Superfrost Plus charged glass slides (Fisher Scientific 12-550-15) and allowed to dry overnight at room temperature. A hydrophobic barrier was drawn around the sections using an ImmEdge Pen (Vector Laboratories H-4000). Sections were prepared for hybridization by incubation in Protease IV (ACDBio 322336) for 30 min at 40°C. Sections were labeled with probes listed in Table 2 according to manufacturer’s instructions for the RNAscope Fluorescent Multiplex Reagent Kit v1 (ACDBio 320850). After hybridization and labeling, sections were allowed to dry before sealing with ProLong Gold antifade reagent with DAPI (Invitrogen P36935). Images of RNAscope labeled sections were collected on a Zeiss AxioImager Z1 widefield epifluorescence microscope.

**Table 2.** ACDBio RNAscope probes

Probe target	ACDBio catalog number
*Gpr4*	427941
*Slc17a6*	319171-C2
*Tph2*	318691-C2
*Slc32a1*	319191-C3
*Pecam1*	316721-C2

### Image analysis

For both widefield and confocal images, Z-stacks were collected through the thickness of the tissue and collapsed into maximum intensity projections for image processing. Background was corrected using the MOSAIC Suite background correction tool in Fiji. Brightness and contrast were adjusted using Fiji in order to maximize signal and minimize background. Images from sections from the same brain region were adjusted by an equal amount. For certain images, adjacent frames were stitched together using the built-in stitching tool in Fiji to generate larger composites for figures.

### Cell counts and analysis

Serial sections (1:3 series) through the rostrocaudal extent of the RTN and caudal raphe were analyzed during all IHC experiments. At least three representative sections from each animal were taken for the thalamus, lateral septum, and dorsal raphe. Images were acquired using an epifluorescence microscope (Zeiss AxioImager Z1) equipped with Neurolucida software. Labeled cells were counted and aligned for averaging according to defined anatomical landmarks (Paxinos and Franklin, 2001). Tracings were exported to NeuroExplorer software (MBF Bioscience) for analysis of cell numbers within the ventral brainstem and 2D plots of marker locations on section traces.

### Stereotaxic injection of adeno-associated virus (AAV) into the RTN

*Nmb*^Cre/+^ mice contain an IRES-Cre knock-in to the endogenous *Nmb* locus so that Cre expression mirrors NMB expression in the RTN. Details on generation and characterization of this line can be found in Souza et al. (2023). Adult *Nmb*^Cre/+^;*Gpr4*^HA/HA^ mice (8–12 weeks old) were anesthetized with ketamine/dexmedetomidine HCl (100 mg kg^−1^ and 0.2 mg kg^−1^, i.p.), mounted in a stereotaxic apparatus and maintained at 37°C with a servo-controlled heating pad. After craniotomy, a pipette filled with AAV2-DIO-mCherry under the control of the human synapsin promoter (VectorBuilder, diluted to ∼2 × 10^9^ transducing units per ml) was inserted at coordinates ∼1.4 mm lateral to midline, 1.4 mm caudal to lambda, and 5.0–5.4 mm ventral to the pial surface of the cerebellum. In addition to stereotaxic coordinates, we recorded antidromic field potentials elicited by stimulating the mandibular branch of the facial nerve to locate the position of the facial motor nucleus more precisely. For unilateral injections in the RTN, the tip of the injection pipette was positioned 100 μm below the facial motor nucleus, and at five rostrocaudally aligned sites along the facial motor nucleus, separated by 200 μm. The glass injection pipette was connected to an electronically controlled pressure valve (Picospritzer II) and brief pressure pulses (3–6 ms) were used to inject 100–150 nl of virus at each site. After surgery, mice were treated with ketoprofen (4 mg/kg, subcutaneously) and allowed to recover on a heated pad. At least 4 weeks elapsed after virus injection before mice were killed and tissue was collected for subsequent analysis.

## Results

**Figure 1.**
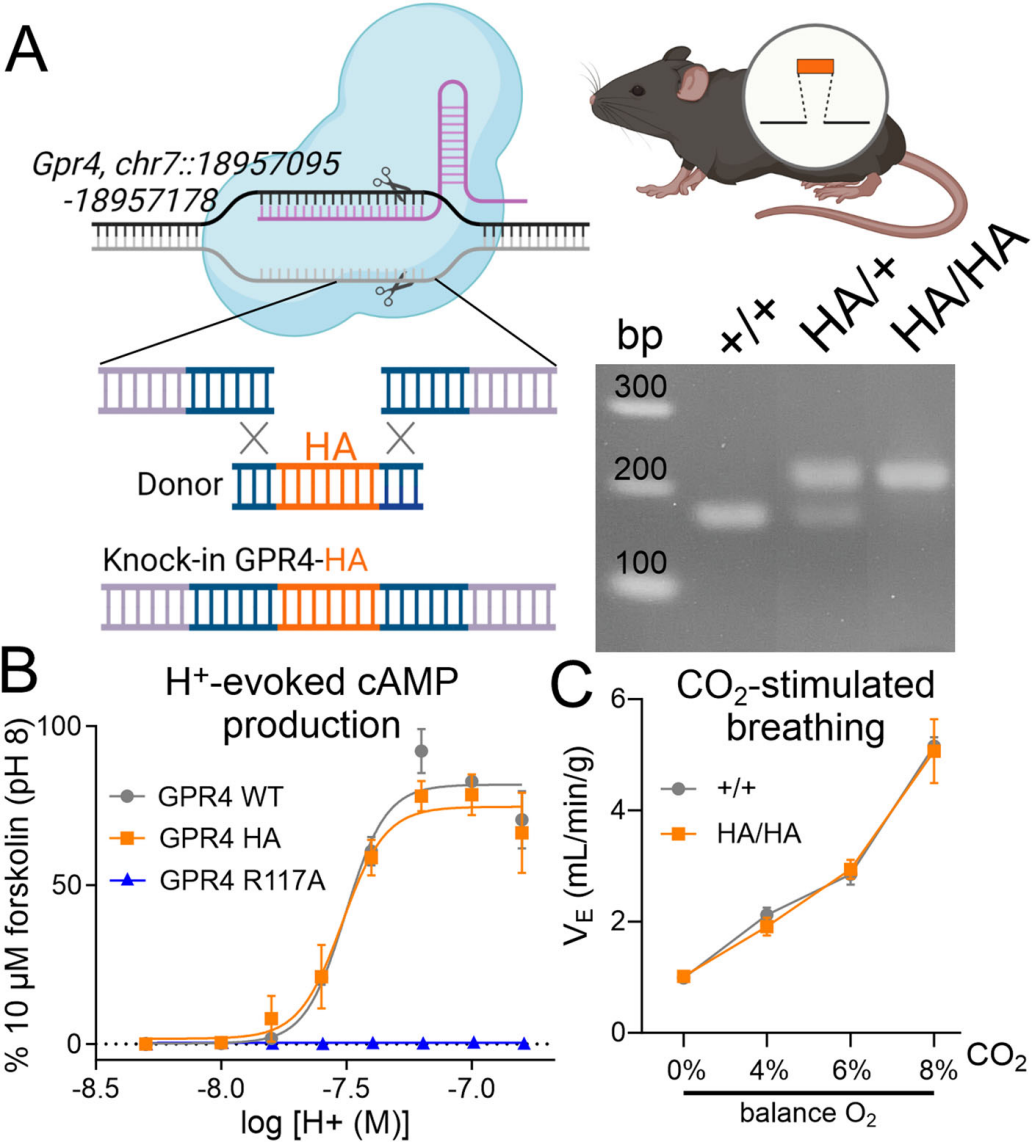
Incorporating an HA epitope tag into GPR4 does not alter receptor function in vitro or the hypercapnic ventilatory reflex in vivo. ***A***, Illustration of the CRISPR/Cas9 knock-in strategy, generated in BioRender, and a representative agarose gel of the diagnostic PCR used to detect the HA tag at the C-terminal end of GPR4. ***B***, Activation by acidification of wild-type and HA-tagged GPR4 in HEK293T cells detected using the luminescent GloSensor assay for cAMP production (normalized to pH-independent, forskolin-activated cAMP production). Note that acidification does not increase cAMP in cells expressing a nonsignaling GPR4(R117A). ***C***, Minute ventilation of *Gpr4*^HA/HA^ and wild-type *Gpr4*^+/+^ mice assessed by whole body plethysmography.

A CRISPR/Cas9 knock-in strategy was used to incorporate an HA tag sequence to the 3′ end of the *Gpr4* coding region at the endogenous locus in the mouse genome (Fig. 1*A*). The addition of this nonapeptide epitope tag (YPYDVPDYA-STOP, preceded by a four-residue GSSG linker) to the extreme C terminus of GPR4 has no effect on pH-dependent cAMP production by GPR4 receptors transiently transfected in HEK293T cells (Fig. 1*B*). In addition, by whole animal plethysmography, GPR4^HA/HA^ knock-in animals demonstrate a normal hypercapnic ventilatory response (HCVR), in comparison with their wild-type GPR4^+/+^ littermates (Fig. 1*C*). Thus, HA-tagged GPR4 retains normal function in these two established assays, in vitro and in vivo.

Target brain regions chosen for assessing GPR4 protein expression using the HA knock-in tag were determined based on previous studies examining *Gpr4* mRNA expression (Magdaleno et al., 2006; Lein et al., 2007; Wyder et al., 2011; Kumar et al., 2015; Shi et al., 2017; Hosford et al., 2018) as well as on our own *Gpr4* mRNA expression screen throughout the brain. For each region described below, we present expression of both GPR4 mRNA and protein.

### Retrotrapezoid nucleus (RTN)

**Figure 2.**
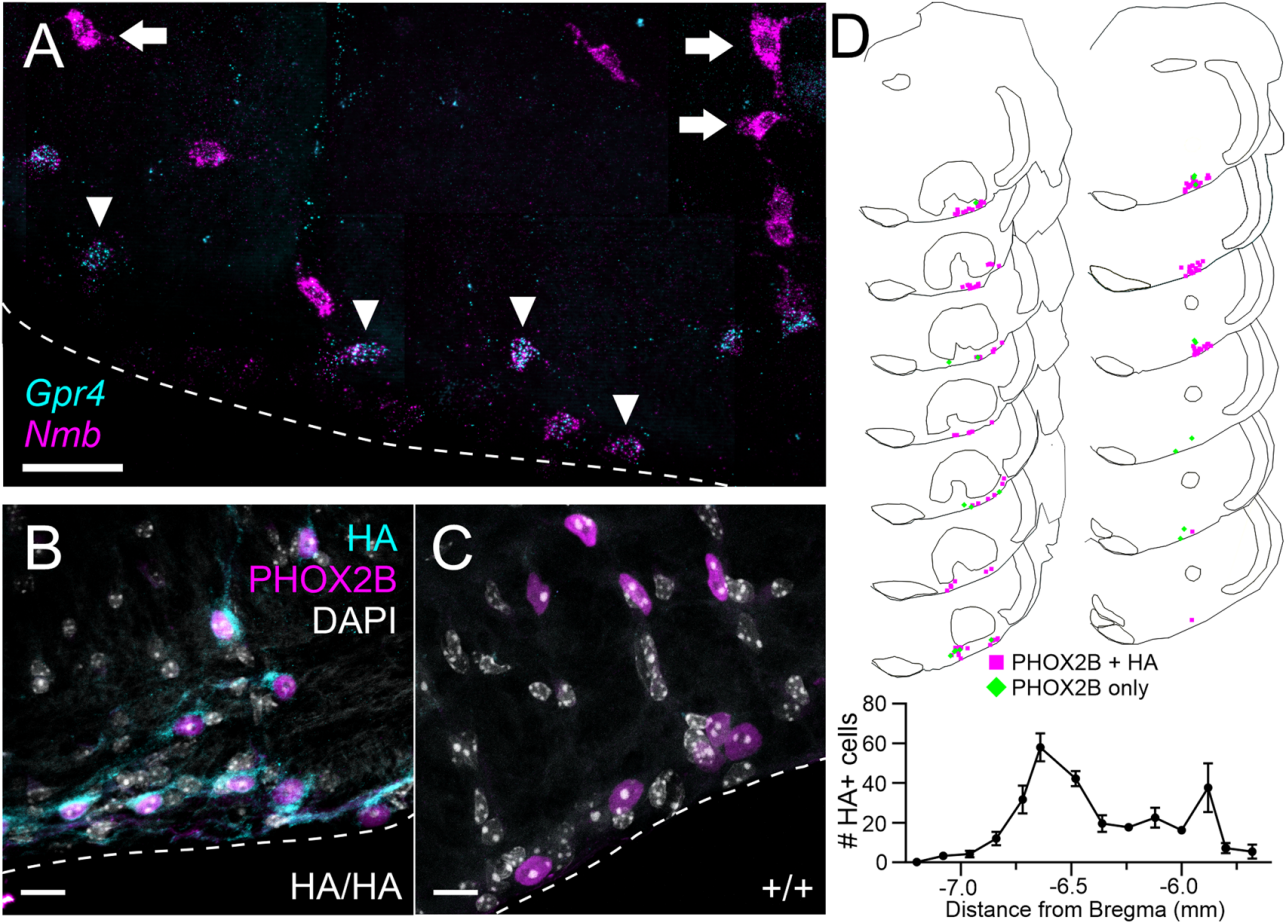
GPR4 transcript and protein expression in the retrotrapezoid nucleus (RTN). ***A***, RNAscope multiplex in situ hybridization (ISH) labeling for *Gpr4* and the RTN marker *Nmb* at bregma level −6.48 mm. Arrowheads indicate RTN neurons that coexpress *Nmb* and *Gpr4*; arrows indicate more dorsally located neurons with high levels of *Nmb* that do not express *Gpr4* (Shi et al., 2017). ***B***, ***C***, HA immunostaining in the RTN of *Gpr4*^HA/HA^ (***B***) and wild-type *Gpr4*^+/+^ (***C***) mice; RTN neurons are identified by expression of PHOX2B. ***D***, Representative maps of PHOX2B+/HA+ cells and PHOX2B-only cells through the rostrocaudal extent of the RTN (top, bregma −5.8 to −7.08), and average distribution of HA+ cells through the RTN (bottom). Data were averaged (±SEM) from four mice; scale bar, 50 µm.

RTN neurons are required for CO_2_/H^+^-evoked respiratory chemoreflexes (Guyenet et al., 2019; Gonye and Bayliss, 2023; Souza et al., 2023), and they can be identified within the parafacial region of the mouse rostral ventrolateral medulla by their expression of PHOX2B, and more specifically by expression of Neuromedin B (Nmb; Shi et al., 2017). *Nmb*-expressing RTN neurons express high levels of *Gpr4* transcript (Kumar et al., 2015; Shi et al., 2017; Hosford et al., 2018). Indeed, in wild-type C57BL6/J mice (Fig. 2*A*), *Gpr4* expression can be clearly localized to *Nmb*^+^ (RTN) neurons in the parafacial region of the medulla (*arrowheads*); as described earlier (Shi et al., 2017), some dorsally located neurons with especially high levels of *Nmb* do not express *Gpr4* (arrows). In this region, specific HA staining is observed in the cell bodies of PHOX2B^+^ neurons and in their processes along the ventral surface of the medulla (Fig. 2*B*). HA-positive signal is only evident in GPR4^HA/HA^ animals, not their GPR4^+/+^ littermates (Fig. 2*B*,*C*). The rostrocaudal distribution of HA^+^ cells in this region closely mirrors that of RTN neurons (Fig. 2*D*; Lazarenko et al., 2010; Kumar et al., 2015; Shi et al., 2017), and the percentage of PHOX2B^+^ cells that are immunoreactive for HA (74.6 ± 6.4, *n* = 4 animals) is comparable to that previously reported for the percentage of PHOX2B-expressing neurons that contain *GPR4* transcripts (∼70%; Kumar et al., 2015).

### Caudal raphe nuclei

**Figure 3.**
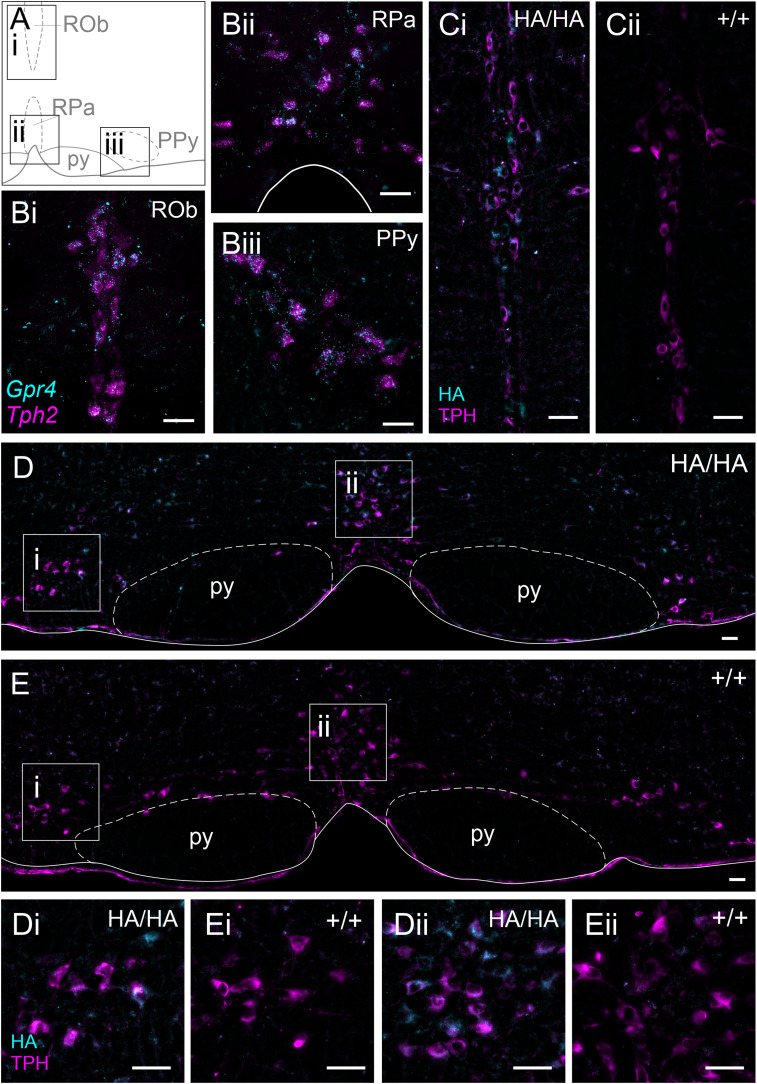
GPR4 transcript and protein expression in the caudal raphe. ***A***, Representative diagram of three caudal raphe nuclei and notable landmarks, based on the Paxinos and Franklin atlas, bregma −6.64 mm. ROb, raphe obscurus; RPa, raphe pallidus; PPy, parapyramidal nucleus; py, pyramidal tract. ***Bi–iii***, RNAscope ISH labeling of *Gpr4* expression in serotonergic raphe neurons identified by *Tph2* expression. ***Ci,ii***, HA staining in the raphe obscurus serotonergic (TPH^+^) nucleus of *Gpr4*^HA/HA^ (***i***) and wild-type *Gpr4*^+/+^ (***ii***) mice. ***D***, ***E***, HA staining in the parapyramidal (***i***) and raphe pallidus (***ii***) nuclei of *Gpr4*^HA/HA^ (***D***) and wild-type *Gpr4*^+/+^ (***E***) mice. Scale bar, 50 µm.

**Figure 4.**
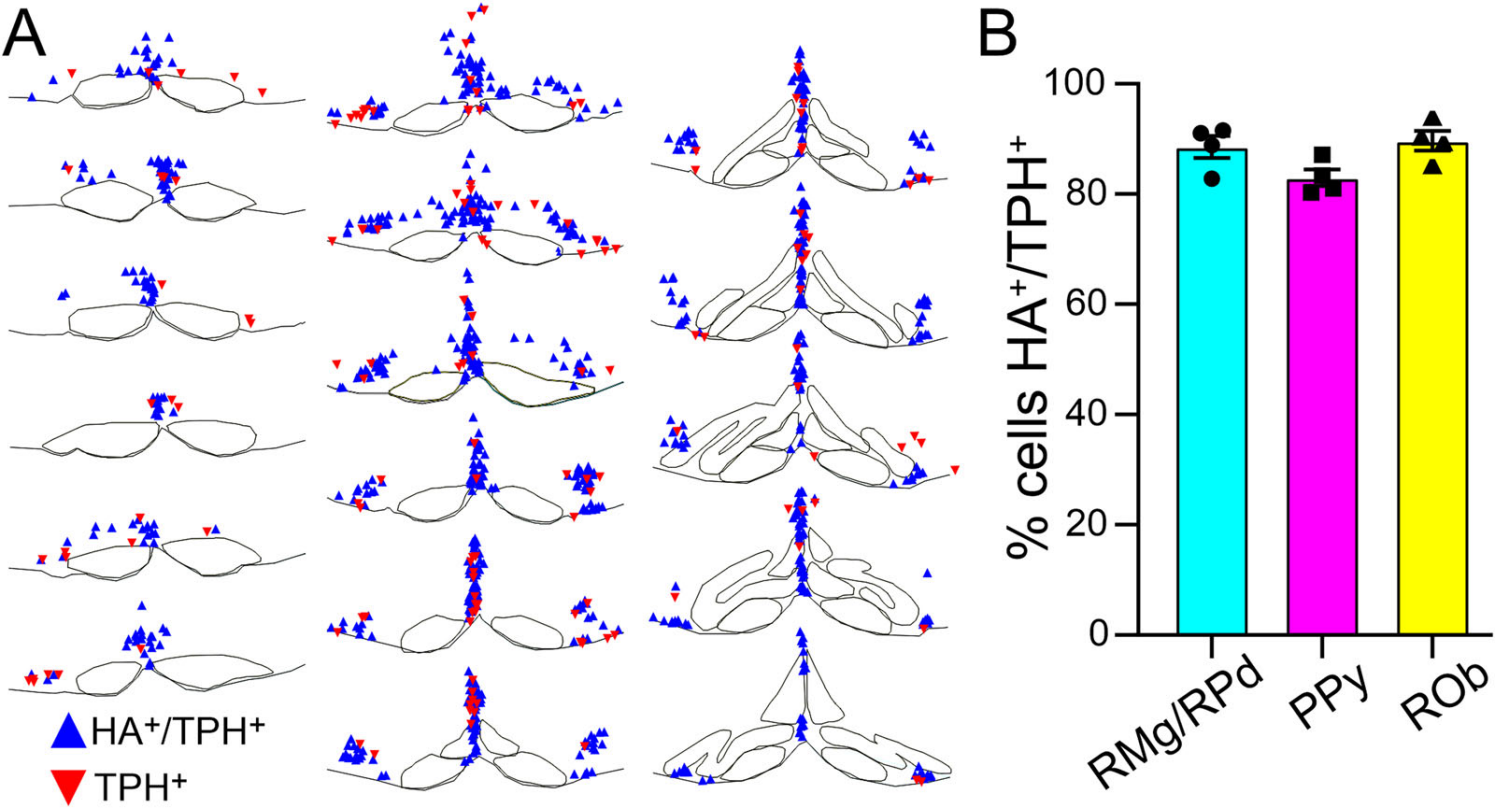
Location and proportion of serotonergic caudal raphe neurons that express GPR4. ***A***, Representative maps of HA^+^/TPH^+^ cells and TPH^+^ cell locations through the caudal raphe, bregma levels −5.80 to −7.12. ***B***, Average percentage of TPH^+^ cells that are also HA^+^ (% HA^+^/TPH^+^) within ROb (33 ± 4 TPH^+^ cells/section), RPa/RMg (23 ± 3 TPH^+^ cells/section), and PPy (26 ± 3 TPH^+^ cells/section). Data were averaged (±SEM) from four mice.

The caudal raphe nuclei (Fig. 3*A*) have been proposed as an important cell group contributing to central respiratory chemoreception (Hodges and Richerson, 2008, 2010; Corcoran et al., 2009), and previous work has localized *Gpr4* transcripts to serotonergic raphe neurons (Kumar et al., 2015; Hosford et al., 2018). Likewise, we find that *Gpr4* mRNA expression can be observed in serotonergic *Tph2*^+^ cells throughout the raphe magnus (RMg), raphe pallidus (RPa), raphe obscurus (ROb), as well as the parapyramidal (PPy) raphe (Fig. 3*B*). Using these mRNA expression results as a guide, we assessed HA staining patterns in the same raphe nuclei. Indeed, HA immunoreactivity is detected in all subdivisions of the serotonergic raphe that display *Gpr4* transcript (Fig. 3*C–E*). Within the cell, HA staining labels the soma and large proximal neurites. The spatial distribution of HA^+^/TPH^+^ neurons throughout the range of the caudal raphe nuclei (Fig. 4*A*) mirrors transcript localization. Overall, HA staining is detected in ∼90% of serotonergic (TPH^+^) neurons throughout these subdivisions of the caudal raphe (Fig. 4*B*).

### Median and dorsal raphe

**Figure 5.**
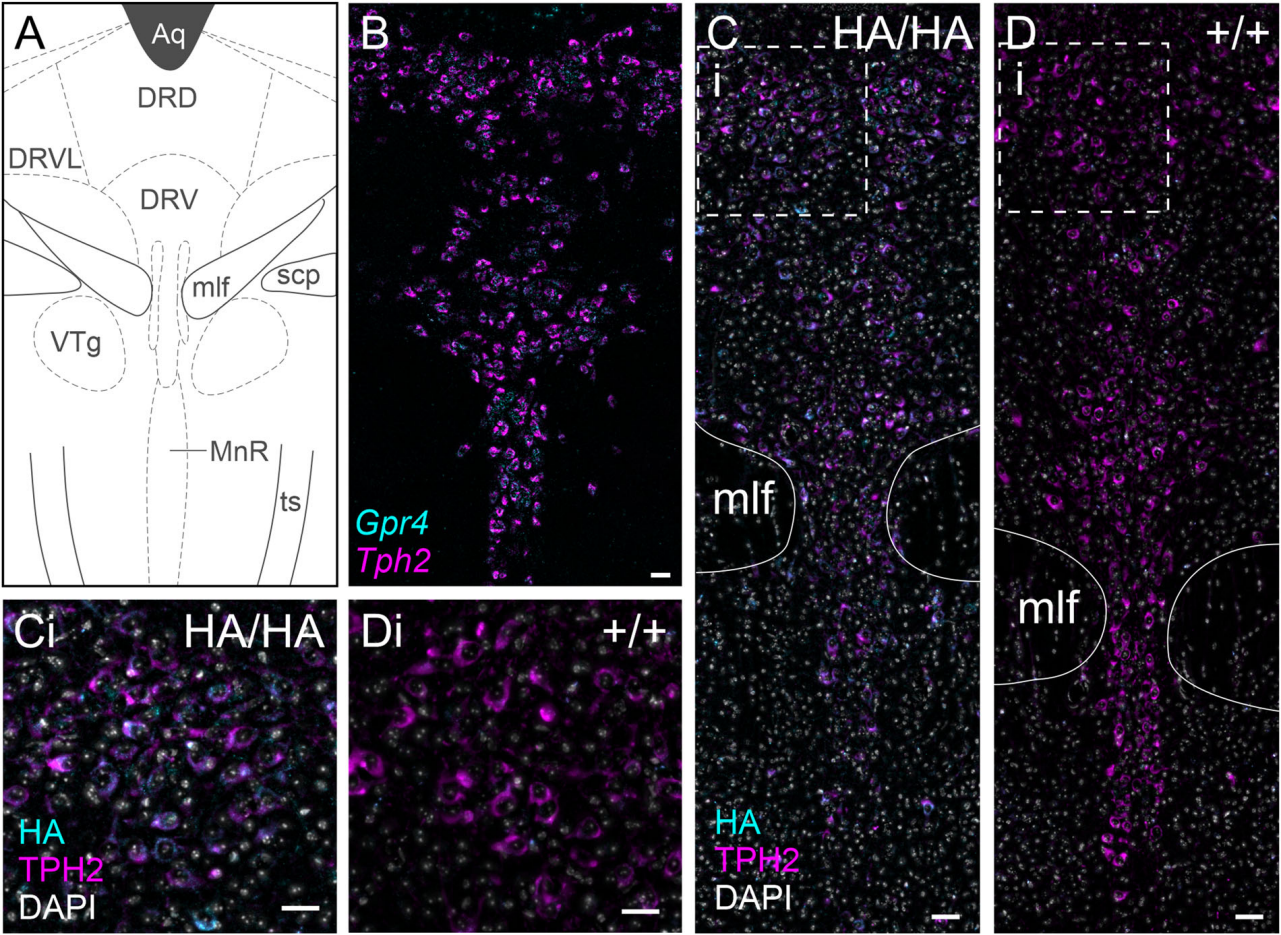
GPR4 transcript and protein expression in the dorsal and median raphe. ***A***, Representative diagram of key nuclei and landmarks at bregma −4.84 mm, including the nuclei of the dorsal and median raphe, according to the Paxinos and Franklin atlas. Aq, aqueduct (Silvius); DRD, dorsal raphe nucleus, dorsal part; DRVL, dorsal raphe, ventrolateral; DRV, dorsal raphe nucleus, ventral; mlf, medial longitudinal fasciculus; scp, superior cerebellar peduncle (brachium conjunctivum); VTg, ventral tegmental nucleus; MnR, median raphe nucleus; ts, tectospinal tract. ***B***, RNAscope ISH labeling demonstrating *Gpr4* expression in *Tph2*^+^ cells of the dorsal and median raphe. ***C***, ***D***, GPR4-HA immunolabeling in the dorsal and median raphe of *Gpr4*^HA/HA^ (***C***) and wild-type *Gpr4*^+/+^ (***D***) mice; (***i***) represents section of the image viewed in higher magnification in ***Ci–Di***. Scale bar, 50 µm.

Previous lineage tracing of GPR4 expression using a Cre/fluorescent reporter system described reporter expression in both the median and dorsal raphe (Hosford et al., 2018). The same work also reported *Gpr4* transcript expression in the dorsal raphe region (Fig. 5*A*). We also observed *Gpr4* expression in *Tph2*^+^ cells of both the median and dorsal raphe (Fig. 5*B*). HA staining in the serotonergic subset of dorsal and median raphe neurons directly corresponds to the expression pattern of *Gpr4* transcript and is only observed in HA knock-in mice (Fig. 5*C*,*D*). HA staining is observed mainly in the soma and large neurites.

### Thalamus

**Figure 6.**
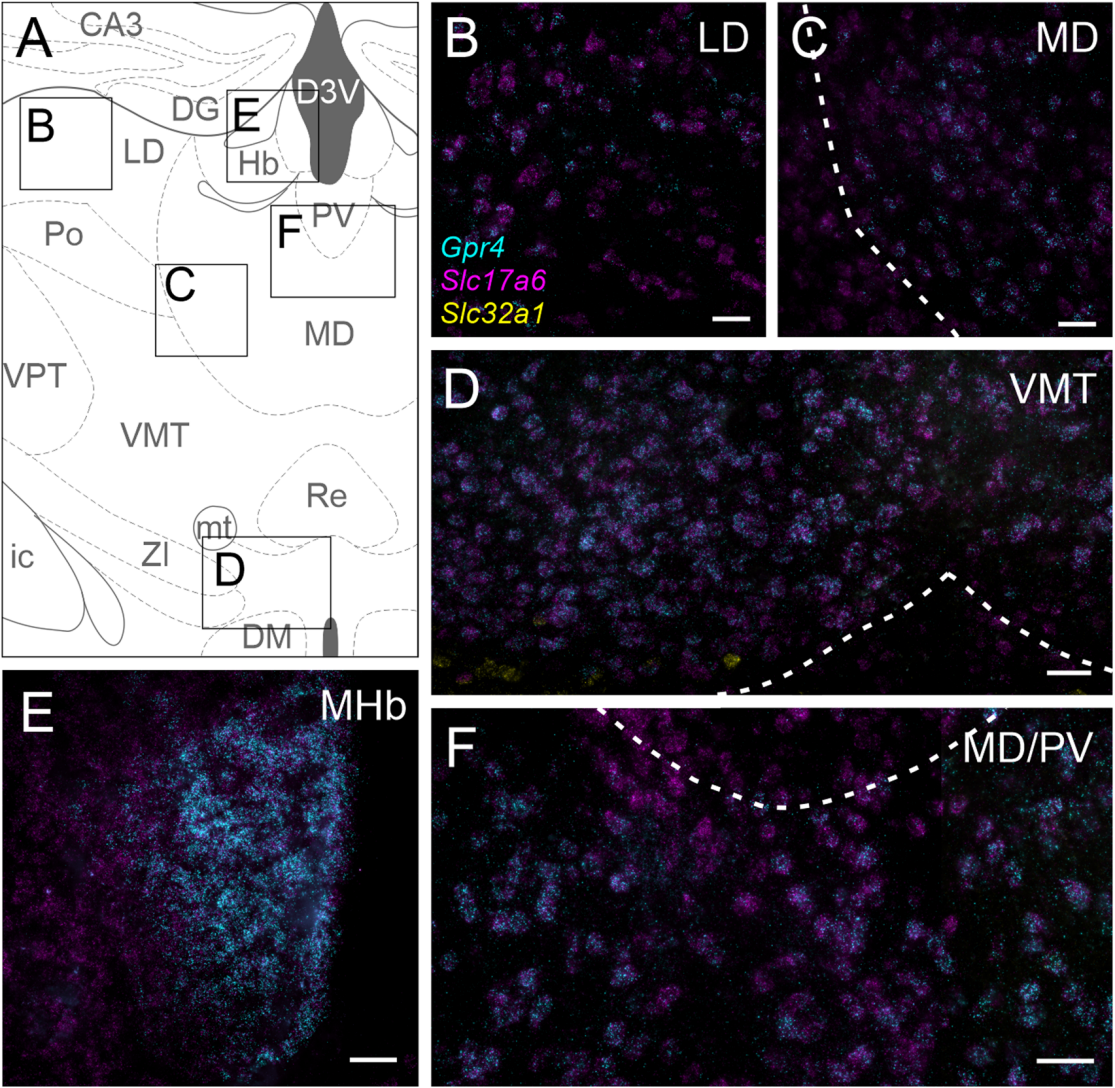
*Gpr4* transcript expression in multiple nuclei of the thalamus. ***A***, Representative diagram of key landmarks and thalamic nuclei at bregma −1.34 mm; inset rectangles represent regions presented in panels ***B–F***. CA3, hippocampus CA3; DG, dentate gyrus; D3V, dorsal third ventricle; LD, laterodorsal thalamus; Hb, habenula; PV, paraventricular thalamus; Po, posterior thalamic group; MD, mediodorsal thalamus; VPT, ventroposterior thalamus; VMT, ventromedial thalamus; Re, reuniens thalamic nucleus; mt, mammillary tract; ic, internal capsule; ZI, zona incerta; DM, dorsomedial hypothalamus. ***B–F***, RNAscope in situ labeling of *Gpr4* expression in glutamatergic (*Slc17a6*) cells of the laterodorsal (***B***), mediodorsal (***C***, ***F***), medial habenula (***E***), and ventromedial (***D***) thalamic nuclei. A few GABAergic (*Slc32a1*) cells visible at the ventral border of VMT appear negative for *Gpr4*. Scale bar, 50 µm.

**Figure 7.**
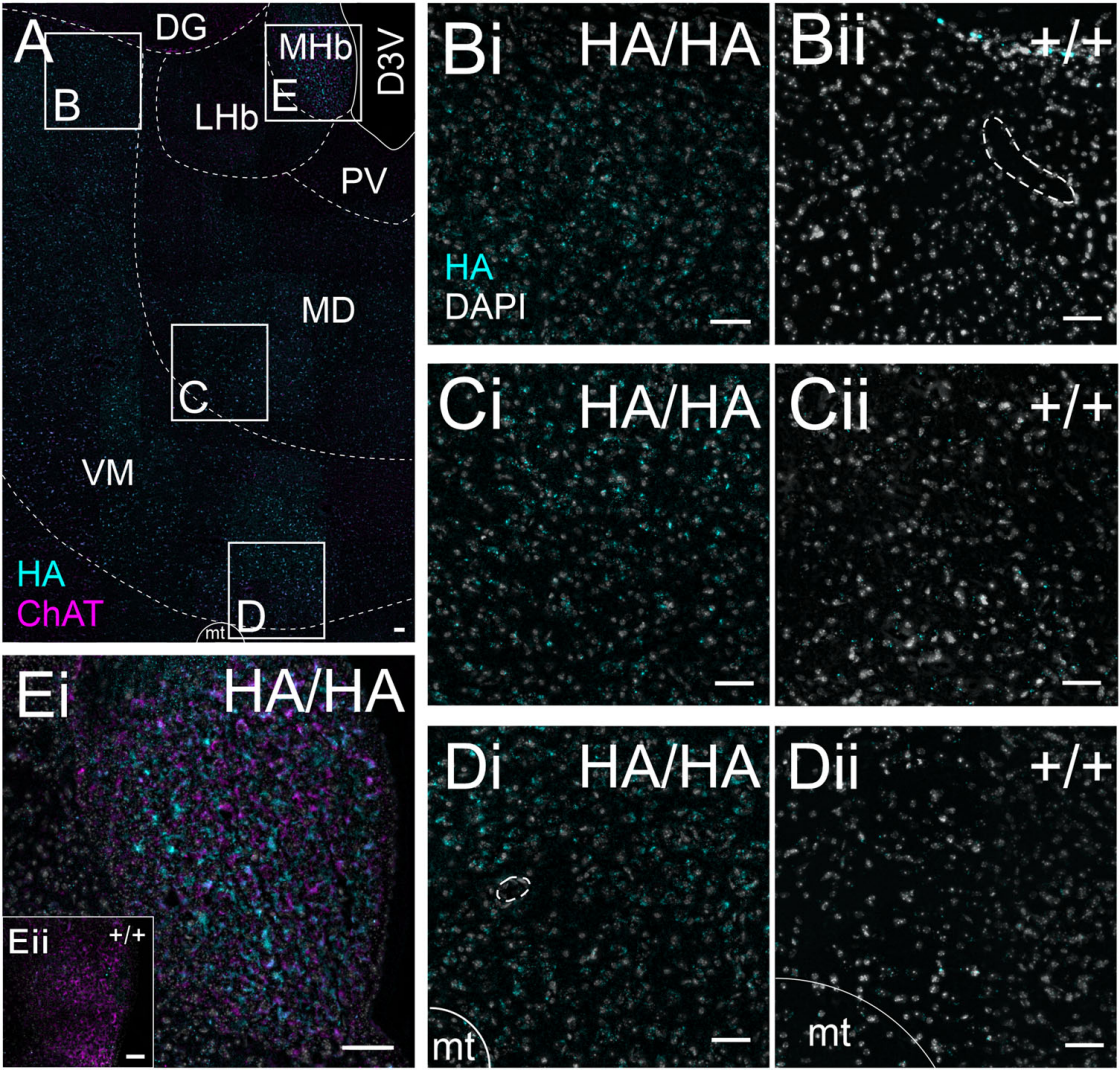
GPR4 protein expression in the thalamus. ***A***, Composite image of the thalamus of a *Gpr4*^HA/HA^ mouse stained for HA and ChAT, with general landmarks delineated by dashed lines. DG, dentate gyrus; D3V, dorsal third ventricle; LD, laterodorsal thalamus; LHb, lateral habenula; MHb, medial habenula; PV, paraventricular thalamus; VM, ventromedial thalamus. ***B–D***, HA staining (with DAPI labeling) in the laterodorsal (***B***), mediodorsal (***C***), and ventromedial (***D***) thalamus of *Gpr4*^HA/HA^ (***i***) and wild-type *Gpr4*^+/+^ (***ii***) mice. ***Ei***, HA and ChAT staining in the medial habenula of *Gpr4*^HA/HA^ and wild-type *Gpr4*^+/+^ mice (***Eii***, inset). Scale bar, 50 µm; dotted lines identify blood vessels (in ***Bii***,***Di***).

**Figure 8.**
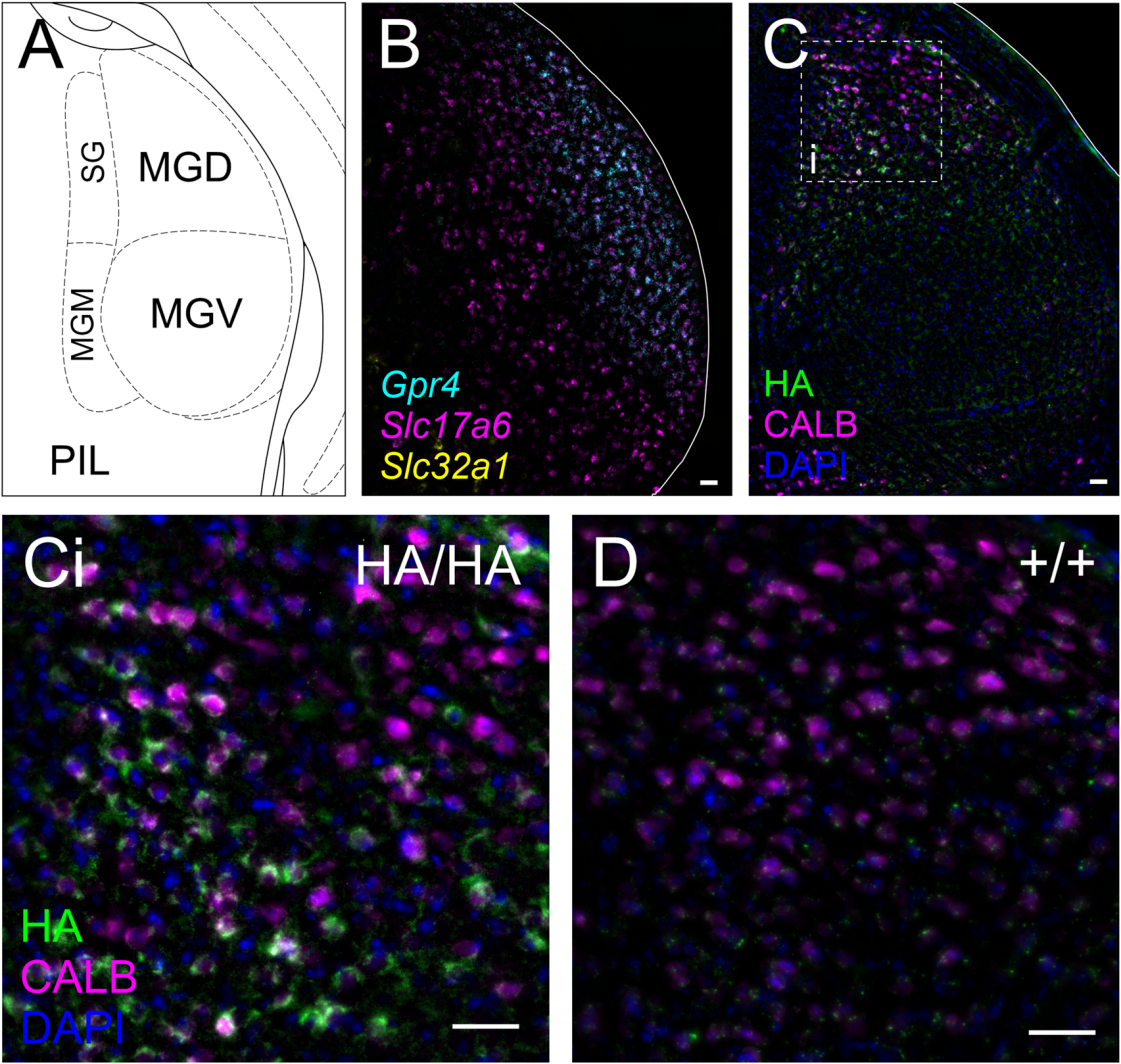
GPR4 transcript and protein expression in the geniculate nucleus. ***A***, Representative diagram of key landmarks around the medial geniculate nucleus at bregma level −3.28 mm. MGD, medial geniculate nucleus, dorsal part; MGV, medial geniculate nucleus, ventral part; MGM, medial geniculate nucleus, medial part; SG, suprageniculate nucleus; PIL, posterior intralaminar thalamic nucleus. ***B***, RNAscope ISH labeling of *Gpr4* expression in the geniculate nucleus, together with markers for glutamatergic (*Slc17a6*) and GABAergic neurons (*Slc32a1*). ***C***, HA staining in the Calbindin B (CALB) expressing cells of the dorsal aspect of the geniculate nucleus of *Gpr4*^HA/HA^ mice; dotted line designates region viewed at higher power in (***Ci***). ***D***, Lack of HA staining in a wild-type mouse at the region analogous to that displayed in (***Ci***).

*Gpr4* transcript can be observed in multiple glutamatergic thalamic nuclei (Fig. 6), in which neurons express the vesicular glutamate transporter 2 (VGlut2, encoded by *Slc17a6*). Similarly, GPR4-HA staining can be noted in the same locations as *Gpr4* transcript throughout the thalamus (Fig. 7*A–E*). In addition, *Gpr4* transcripts are evident in the medial habenula (Fig. 6*E*); in the habenula, HA staining labels the cell bodies of the cholinergic neurons (choline acetyltransferase, ChAT^+^) cells in the ventrolateral division of the nucleus (Fig. 7*E*). The intensity of HA staining varies between individual cells within the habenula, but ChAT^−^/HA^+^ cells were not observed in the sections examined for these studies. A small population of ChAT^+^/HA^−^ cells are also present. In addition to thalamic cell groups described in Figures 6 and 7, high levels of *Gpr4* transcript are observed in the glutamatergic (*Slc17a6*^+^) cells of the dorsal aspect of the medial geniculate nucleus (Fig. 8*A*,*B*). Strong GPR4-HA staining can be also observed in calbindin-expressing cells at the same anatomical location, corresponding to the excitatory neurons identified by *Gpr4* transcript expression and corresponding to cluster of neurons previously identified in the geniculate via single nucleus RNA sequencing (Bakken et al., 2021; Fig. 8*C*,*D*).

### Lateral septum

**Figure 9.**
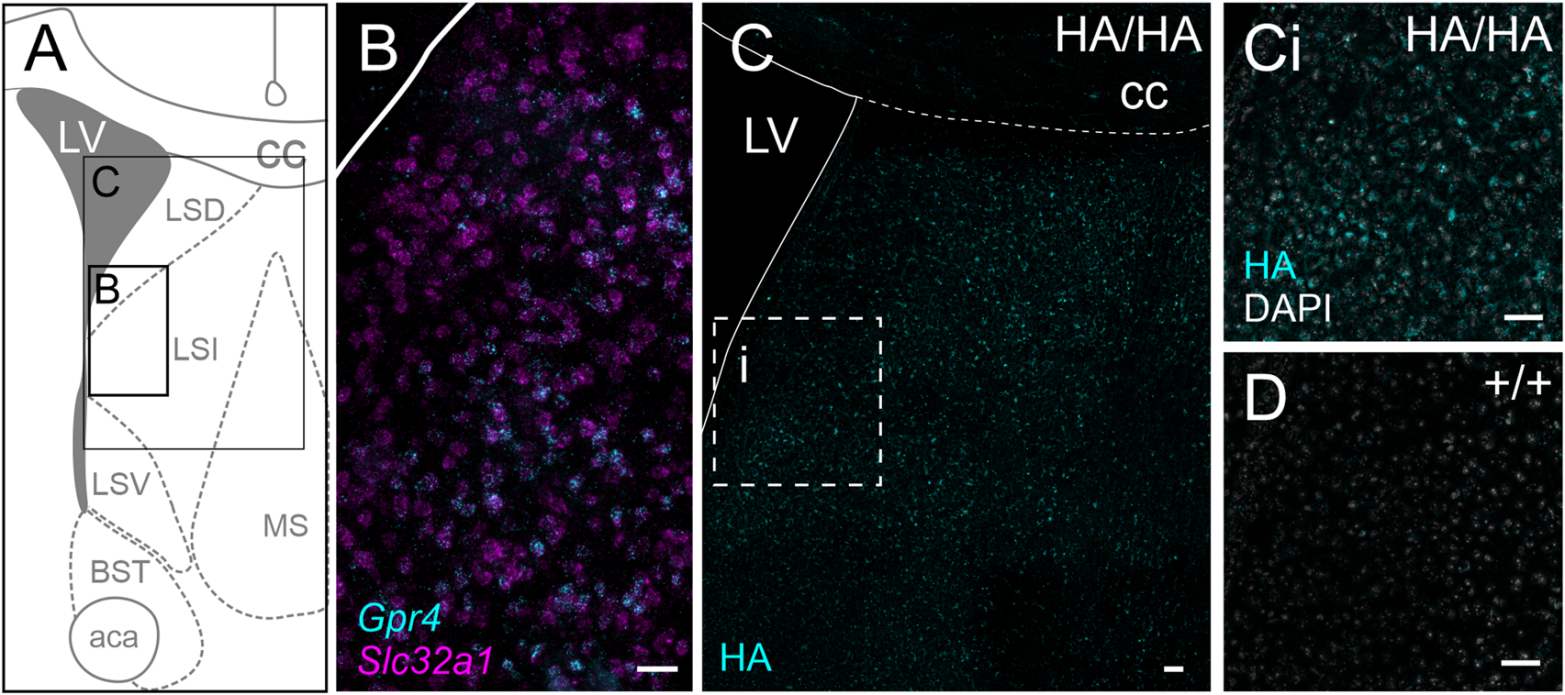
GPR4 transcript and protein expression in the lateral septum. ***A***, Representative diagram of key landmarks around the lateral septum at bregma level 0.38 mm. LV, lateral ventricle; cc, corpus callosum; LSD, dorsal lateral septal nucleus; LSI, intermediate lateral septal nucleus; LSV, ventral septal nucleus; MS, medial septal nucleus; BST, bed nucleus of the stria terminalis; aca, anterior commissure anterior part. ***B***, RNAscope ISH labeling of *Gpr4* expression in GABAergic (*Slc32a1*) neurons of the lateral septum. ***C***, HA staining throughout the lateral septum of a *Gpr4*^HA/HA^ mouse; the indicated region is displayed at higher magnification in (***Ci***). ***D***, Lack of HA staining in a wild-type mouse at a region analogous to that displayed in (***Ci***). Scale bar, 50 µm.

As previously reported (Hosford et al., 2018), *Gpr4* expression can be observed in the GABAergic cells of the lateral septum (marked by expression of the GABA vesicular transporter, Vgat1; *Slc32a1*). Increasing levels of *Gpr4* expression are apparent moving medially from the lateral ventricle toward the intermediate portion of the lateral septum and the medial septum (Fig. 9*A*,*B*). HA staining in GPR4^HA/HA^ mice exhibits the same spatial pattern as observed for transcript expression and is not observed in wild-type controls (Fig. 9*C*,*D*). These protein expression results also match previous lineage tracing results (Hosford et al., 2018). In the lateral septum, HA staining is mainly observed in the cell bodies and large proximal neurites.

### Vascular endothelium

**Figure 10.**
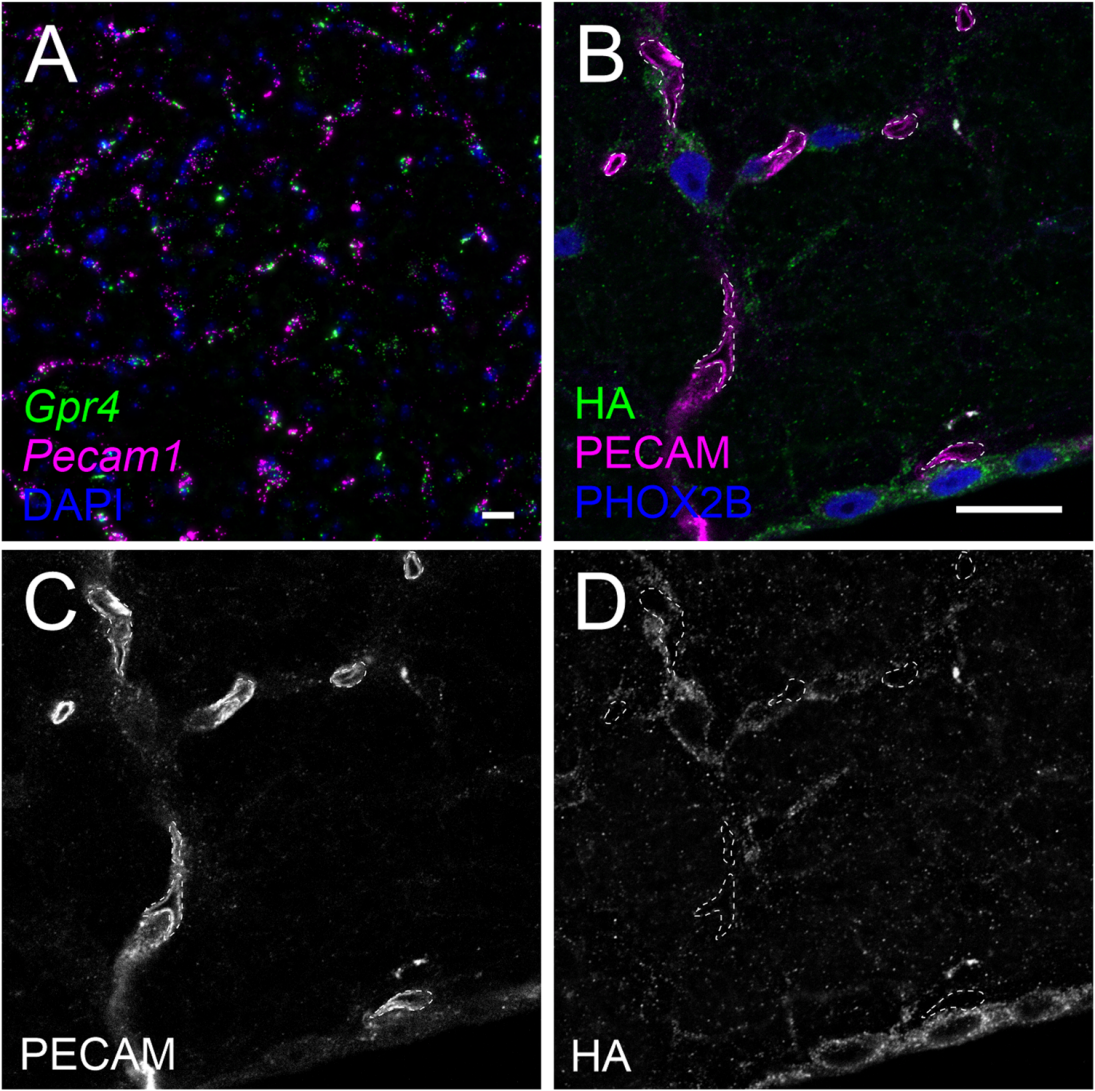
GPR4 transcript, but not protein, is detectable in brain endothelial cells. ***A***, RNAscope in situ labeling of *Gpr4* expression in endothelial (*Pecam1*^+^) cells in the wild-type mouse brainstem. ***B***, ***C***, HA staining in RTN neurons from a *Gpr4*^HA/HA^ mouse, with adjacent small vessels labeled by PECAM expression; vessel cross sections (walls and lumen) are delineated by dashed lines. ***C***, PECAM staining within vessel boundaries. ***D***, HA staining located outside of PECAM^+^ vessels. Scale bar, 25 µm.

*Gpr4* transcript can be detected in brain vascular endothelium, where non-neuronal *Gpr4* labeling in the brainstem overlays strikingly with labeling for the endothelial marker, *Pecam1* (Fig. 10*A*). However, HA labeling is undetectable in PECAM^+^ vascular endothelial cells in sections from GPR4^HA/HA^ mice (Fig. 10*B*,*D*).

### RTN: processes, projections, and terminal fields

**Figure 11.**
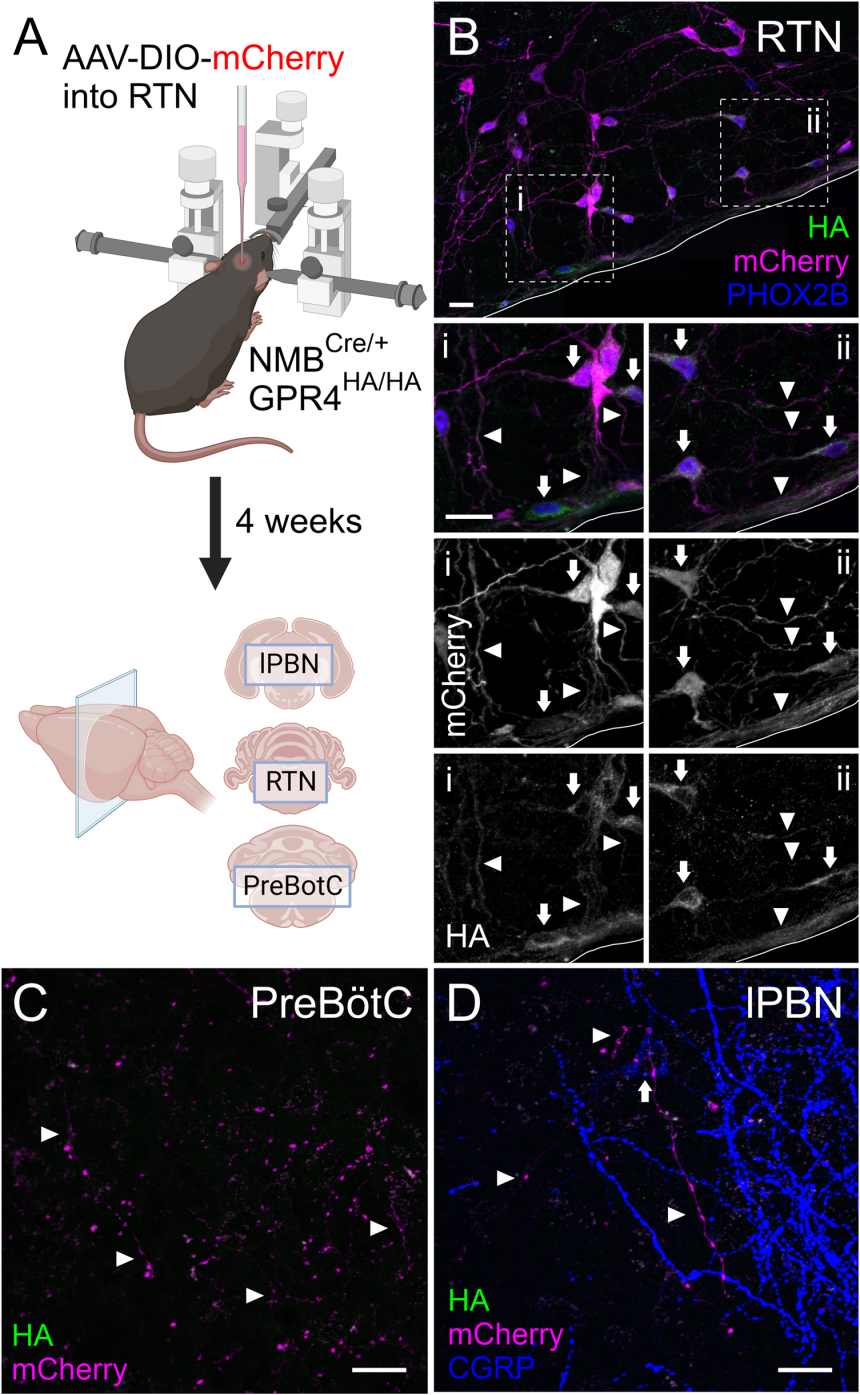
GPR4 is evident in the processes of Nmb-expressing RTN neurons but undetectable at terminals in the preBötC or lPBN. ***A***, Illustration of the viral expression and immunostaining strategy for assessing GPR4 protein expression in processes and terminals of NMB neurons in the RTN area, lPBN, or PreBötC, generated in BioRender. ***B***, HA staining in mCherry labeled neurons in the RTN area of an *Nmb*^Cre/+^; *Gpr4*^HA/HA^ mouse injected with AAV-DIO-mCherry with high power images of areas bounded by dotted lines (***i,ii***); arrows denote staining in RTN cell bodies, arrowheads denote staining in RTN processes. ***C***, mCherry labeled terminals in the preBötC area (bregma −7.48 mm). ***D***, mCherry labeled terminals in the lPBN (denoted by CGRP staining). Scale bar, 25 µm.

We further examined the subcellular organization of HA labeling in the RTN. We leveraged the cell-specific expression of neuromedin B by RTN neurons in the parafacial region of the mouse brainstem (Shi et al., 2017) to specifically label those neurons by injecting an mCherry-expressing, Cre-dependent AAV into that region of *Gpr4*^HA/HA^ animals crossed with an Nmb-Cre mouse (Souza et al., 2023). We then examined HA expression in the mCherry^+^ (i.e., Nmb-expressing) neuronal somata and processes in the RTN itself, as well as in RTN-derived fibers at two previously described RTN projection targets, the preBötzinger complex (preBötC) and the lateral parabrachial nucleus (lPBN; Fig. 11*A*). At the RTN level, HA staining can be observed in mCherry-expressing cell bodies and neuronal processes within the nucleus, as well as in their projections along the ventral medullary surface (Fig. 11*B*). In both the preBötC and lPBN regions, however, we were unable to detect HA staining in mCherry^+^ projections from the RTN (Fig. 11*C*,*D*).

## Discussion

In this study, we leveraged CRISPR/Cas9 genome editing to introduce a small epitope tag sequence into the genomic locus of *Gpr4* to enable detection of GPR4 protein expressed endogenously in the brain. Introduction of the small (13 aa) linker-HA cassette onto the C terminus of GPR4 had no apparent effect on GPR4-mediated signaling in vitro or on CO_2_-stimulated breathing in vivo, two functions for which GPR4 expression is necessary. Examination of HA staining in the brain regions shown to express *Gpr4* transcript reveals detectable GPR4 protein expression in neurons from all loci displaying transcript expression. Thus, these data yield an independent validation of GPR4 protein expression in the mouse brain, confirming a relatively restricted expression of this pH-sensitive GPCR to several neuronal nuclei and providing a new resource to examine GPR4 expression in other tissues where its function has been implicated.

GPR4 expression is not limited to only glutamatergic or GABAergic neuronal populations. GPR4 transcript and protein are present in the GABAergic (*Slc32a1*^+^) neurons of the lateral septum, the glutamatergic (*Slc17a6*^+^) neurons of the thalamus and RTN, and also the cholinergic (ChAT^+^) neurons of the habenula and the serotonergic (TPH^+^) neurons of multiple raphe nuclei. The presence of GPR4 in cells representing a wide variety of molecular signatures indicates a possible role of the receptor in multiple contexts, respiratory or otherwise. In the RTN specifically, GPR4 expression is known to be necessary for a normal HCVR; a blunted HCVR is observed in GPR4 global knock-out mice, and selective reintroduction of GPR4 protein expression into RTN neurons of GPR4 knock-out mice is sufficient to restore the HCVR to the wild-type level (Kumar et al., 2015). Although GPR4 is also expressed in the caudal raphe nuclei, its presence is not required for the activation of serotonergic neurons by CO_2_ (as assessed by Fos expression after an acute CO_2_ challenge) so it may not play a role in manifesting their CO_2_ sensitivity, at least under the conditions tested (Kumar et al., 2015; Hosford et al., 2018). The dorsal and median raphe are critical for CO_2_-induced arousal from sleep (Buchanan and Richerson, 2010; Smith et al., 2018). It is possible that GPR4 expression by these neurons may mediate, at least in part, the direct sensation of CO_2_ by the DR/MR to promote arousal.

Many of the nuclei shown to express GPR4 in this study have been implicated in the manifestation and control of anxiety. CO_2_ is a powerful anxiogenic stimulus in rodents and humans that causes rapid and pronounced autonomic arousal and emotional distress (Bailey et al., 2005, 2011; Poma et al., 2005; Ziemann et al., 2009; Vickers et al., 2012). Chemo- and optogenetic activation of the GABAergic neurons in the lateral septum, in the same area as the cells found to express GPR4 in this study, induces anxiety behaviors in mice (Anthony et al., 2014; Rizzi-Wise and Wang, 2021; Wang et al., 2023). It is possible that the expression of GPR4 by these anxiety-initiating neurons in the lateral septum may mediate some of the anxiogenic effects of CO_2_. The potential role of GPR4 in the medial habenula is more ambiguous. The MHb has been shown to be crucial for anxiety and fear responses, but the specific pathways mediating its role in mood regulation are uncharacterized (Lee et al., 2019; Roy and Parhar, 2022). Gross ablation of the MHb, electrolysis of MHb efferents, or inhibition of MHb neuron firing leads to increased anxiety behaviors and increased circulating corticosterone (Murphy et al., 1996; Kobayashi et al., 2013; Mathuru and Jesuthasan, 2013; Jacinto et al., 2017; Cho et al., 2020). Ablation of the cholinergic neurons of the ventral portion of the MHb, which are the cells shown to express GPR4, leads to increased fear behavior and higher baseline anxiety (Zhang et al., 2016; Lee et al., 2019). It is unclear how activation of the MHb by GPR4 during hypercapnia would lead to the increased anxiety and freezing behavior observed, given the seemingly contradictory observations noted in ablation studies; perhaps GPR4 signaling inhibits the activity of cholinergic MHb neurons. Additionally, there is significant crossover between efferents and afferents of the GPR4-expressing forebrain nuclei; for example, both the lateral septum and medial habenula project to the dopaminergic neurons of the ventral tegmental area (VTA), whose activation induces anxiety behaviors (Herkenham and Nauta, 1977; Qin and Luo, 2009; Yamaguchi et al., 2013; Anthony et al., 2014; Namboodiri et al., 2016; McLaughlin et al., 2017; Rizzi-Wise and Wang, 2021; Besnard and Leroy, 2022; Gouveia and Ibrahim, 2022; Qi et al., 2022; Wang et al., 2023). It is possible that GPR4 acts more broadly to tune the overall anxiety system during hypercapnia and not in activating distinct nuclei. Anxiety phenotypes have not yet been reported in GPR4 knock-out mice.

### Limitations and caveats

We identified HA immunoreactivity in all neuronal populations where *Gpr4* mRNA was found, a correspondence suggesting faithful protein translation of the transcript and detection of the incorporated epitope tag. However, HA staining was not detected in endothelial cells of blood vessels, despite the clear presence of transcript in those same endothelial cells. From these results, it is not possible to rule out GPR4 expression in vascular endothelial cells; it is conceivable that the knock-in of a single HA tag does not provide enough sensitivity to detect GPR4 at the relatively low levels of transcript present in blood vessels. While GPR4 protein can be abundantly detected via HA labeling at the cell body and in long ventral surface dendrites of RTN neurons, it was not visible in terminals at two RTN targets. Again, this observation could be due to endogenous protein expression being too low to detect without further amplification of immunostaining signal or incorporation of multiple HA epitopes in sequence (e.g., 3xHA). Thus, these data do not unambiguously indicate that GPR4 protein is absent from vessels, terminals, or more long-distance projections.

Together, this work reveals a relatively restricted expression pattern of neuronal GPR4 expression in mouse brain that is nevertheless associated with a variety of cell types (i.e., glutamatergic, GABAergic, serotonergic, cholinergic). Aside from its demonstrated contribution to respiratory chemosensitivity via RTN neurons (Kumar et al., 2015; Hosford et al., 2018), the role of GPR4 in additional CO_2_/H^+^-sensitive processes (e.g., arousal, anxiety) mediated by other GPR4-expressing neurons remains to be determined. Finally, the availability of this mouse line will allow detection of GPR4 expression in other peripheral tissues where its role in (patho)physiological processes has been suggested.

## Synthesis

Reviewing Editor: Michael Michaelides, NIDA-NIH Decisions are customarily a result of the Reviewing Editor and the peer reviewers coming together and discussing their recommendations until a consensus is reached. When revisions are invited, a fact-based synthesis statement explaining their decision and outlining what is needed to prepare a revision will be listed below. The following reviewer(s) agreed to reveal their identity: NONE.

Reviewer 1

The authors of manuscript eN-NWR-0002-24 report the development and characterization of a novel HA-tagged GPR4 transgenic mouse line. The transgene design involved using electroporation of CRISPR reagents into mouse embryos to affect the C-terminal tagging of GPR4 with a GSSG-HA epitope. Immunofluorescence assays were used to visualize the anti-HA staining patterns with various neuronal markers in brain areas that were already known to express GPR4 transcripts. The analysis and discussion were well-grounded and supported by the data presented.

Abstract

Line 6 = suggest "visualize" instead of "localize"

Line 7 = "protein expression" cannot be verified by mRNA detection, suggest remove "protein"

Line 10 = this statement unclear or unsupported by the data. GPR4 expression "must be restricted to specific cell types" or else it would be seen everywhere/every cell, and the data shown here and by others indicates that it is not in every cell/neuron.

Significance Statement -

This section seemed well written and cogent.

Intro

This section seemed well written and cogent.

Methods -

Line 87 - GloSensor cAMP: it is not clear how the authors are controlling for any cAMP-based activity that might be natively produced by HEK293T in response to pH changes. With the information available (a comparison of "HA-tagged" GPR4 vs "WT" GPR4), it can only be concluded that the two GPR4 versions do not contribute any difference in activity. I suggest that the two existing plasmids be compared to an additional transfection (pcDNA3.1 "empty") that would demonstrate that transfection with GPR4 is necessary to respond to pH changes. Alternatively, the invitro data could be removed and the work can rely on the "Whole body plethysmography" assay.

Line 136 - Please provide these details:

define "TS", is it Tris saline?, indicate the pH of the final solution.

Define FHS (fetal horse serum?)

Line 179 - Please provide these details:

Define "TU"

What is the promoter driving mCherry in the virus?

What is the source of the AAV (Where/How was it produced?)

Results

Line 194 - please see and address the concerns previously expressed above (line 87) with regard to the in vitro assay.

Line 213 - Just curious, is detection sensitive enough to observe staining in heterozygotes (HA/+)?

Line 217 - Please clarify, what is unit for "n=4" (e.g., #mice, or number sections observed)

Line 242 - For this reviewer, distinguishing the staining (HA vs CHAT) in Fig 7 was more difficult than distinguishing (HA vs tph2) in Fig 6. It appears that there are significant population of (HA+/CHAT-) and (HA-/CHAT+) cells, but this is not reflected in the text. Please clarify/address this in the text and/or provide single channel images to support the claim.

Line 248 - This is an example of circular reasoning: "HA staining exhibits the same spatial pattern and is specific to mice expressing the HA-tagged receptor".. Perhaps it is intended as "staining observed only in animals carrying the transgenic allele but not in wildtype". In any case, please restate/clarify.

Line 258 - Please see and address the concerns previously expressed above (line 179) with regard to the details about the AAV vector.

Line 258 - Please provide details about the source (or resource ID) of this Nmb-Cre driver line.

Discussion

END - this discussion, limitations, caveats section finishes very well. A pleasure to read.

Comments on the Figures/Figure Legends

FIG 1 - these look like images from Bio-render... check legend for acknowledgement.

Line 578 - FIG 1B needs a better explanation for the y-axis label

Line 579 - please see and address the concerns previously expressed above (line 87) with regard to the in vitro assay.

Fig 4 -

Line 599 - figure legend (4A and 4B) is inconsistent with the annotations on the graph, and both need correction/reconciliation. Please clarify the label for the y-axis. Please disclose how many cells were quantified for each group, in addition to the calculated percentage.

Fig 10 -

Line 645 - these graphics appear to be from BioRender, but I do not see an acknowledgement in the legend.

Reviewer 2

Thank you for the opportunity to review the manuscript entitled "Expression of Endogenous Epitope-Tagged GPR4 in the Mouse Brain." The manuscript describes the successful generation and validation of a HA-tagged GPR4 knock-in model in mice. The histological and cell distribution analysis data are convincing, and the functional assessments through pH sensing in cells and respiratory function in response to increasing CO2 levels in vivo suggest retention of the tagged receptor's function compared to the wild-type.

Overall, the manuscript is well-written, and the experiments provide sufficient initial validation of the model. However, the study does not delve beyond establishing the validity of the model to further explore the cellular and molecular biology of the receptor in knock-in mice.

Therefore, I recommend publication of the manuscript as a valuable tool development study. However, there is a minor detail requiring clarification before final approval: the number of male and female mice used in the study. While the Materials and Methods section mentions a total of 6 mice, the specific ratio of males to females remains unclear. Adding a sentence specifying this ratio would be beneficial.
